# 

**Published:** 1854-12

**Authors:** 


					﻿No. VII.]
DECEMBER.
[Vol. X.
BUFFALO
MEDICAL JOURNAL
AND
JHantljh) lieuteiv
or
MEDICAL AND SURGICAL SCIENCE.
AUSTIN FLINT, M. D.,	)
SANFORD B. HUNT, M. D., $ Editors-
BUFFALO, N. Y.:
PUBLISHED BY THOM AS <t LATHROPS.
Commercial Advertiser Buildings.
1854.
Price $2.50 per annum, in advance.
				

